# Early onset age increases the risk of musculoskeletal damage in patients with type 2 diabetes

**DOI:** 10.3389/fendo.2023.1270674

**Published:** 2023-12-08

**Authors:** Biao Zheng, Yongze Zhang, Lingning Huang, Ximei Shen, Fengying Zhao, Sunjie Yan

**Affiliations:** ^1^ Department of Endocrinology, The First Affiliated Hospital, Fujian Medical University, Fuzhou, China; ^2^ Department of Endocrinology, National Regional Medical Center, Binhai Campus of the First Affiliated Hospital, Fujian Medical University, Fuzhou, China; ^3^ Clinical Research Center for Metabolic Diseases of Fujian Province, The First Affiliated Hospital, Fujian Medical University, Fuzhou, China; ^4^ Fujian Key Laboratory of Glycolipid and Bone Mineral Metabolism, The First Affiliated Hospital, Fujian Medical University, Fuzhou, China; ^5^ Diabetes Research Institute of Fujian Province, The First Affiliated Hospital, Fujian Medical University, Fuzhou, China; ^6^ Metabolic Diseases Research Institute, The First Affiliated Hospital, Fujian Medical University, Fuzhou, China

**Keywords:** onset-age, diabetes, osteoporosis, sarcopenia, musculoskeletal damage

## Abstract

**Introduction:**

It’s not clear whether there are differences in musculoskeletal damage and body composition among different age groups of type 2 diabetes. Therefore, the purpose of this study is to analyze the difference between early-onset type 2 diabetes (EOT2D) and non-early-onset type 2 diabetes (NOT2D) in musculoskeletal damage.

**Methods:**

A total of 964 patients with type 2 diabetes mellitus were selected by 1:1 propensity score matching, including 534 males and 430 females, with an average age of 52 ± 7 years and an average course of 10 ± 8.5 years. Bone mineral density and body composition were measured, and combined with biochemical tests, linear regression and binary logic regression were used to analyze the relationship between EOT2D, NOT2D and musculoskeletal damage. In addition, 414 patients with T2DM were selected according to whether they were hospitalized twice or not, and the median follow-up period was 44 months. COX survival analysis further elucidates the relationship between EOT2D, NOT2D and musculoskeletal damage.

**Results:**

Compared with patients with non-early-onset type 2 diabetes, A/G was negatively correlated with the age of onset, and had statistical significance. EOT2D has a higher risk of sarcopenia, osteoporosis and even musculoskeletal damage. With the prolongation of the course of the disease, the risk of muscle mass and/or bone mineral density decrease in EOT2D increases.

**Conclusion:**

EOT2D brings a greater risk of sarcopenia and/or osteoporosis, as well as a higher risk of reduced ASM and BMD. In addition, fat distribution may be more central.

## 1 Introduction

A recent national survey shows, the prevalence of type 2 diabetes in the national survey from 2015 to 2017 was 11.2% (1). According to the data in 2019, the number of elderly diabetic patients in China is about 35.5 million, ranking first in the world, accounting for 25% of the elderly diabetic patients in the world, and showing a rising trend (2). The definition of diabetes high-risk group includes: (1) age greater than or equal to 40 years old, (2) overweight [body mass index (BMI) greater than or equal to 24kg/m^2^] or obesity (BMI greater than or equal to 28kg/m^2^) and/or central obesity (male waist circumference greater than or equal to 90cm, female waist circumference greater than or equal to 85cm), (3) dyslipidemia, (4) family history or prediabetes personal history, etc. (1). Some studies have shown that patients with type 2 diabetes mellitus (T2DM) tend to be younger (3). Age is used as the cut-off value for the type of diabetes, that is, early-onset diabetes (EOD, age ≤ 40 years old) and non-early-onset diabetes (NOD, age > 40 years old) (4). There is no comprehensive view on whether there is a difference between the two different types of diabetes.

Osteoporosis is an age-related disease (5). And some studies have concluded that T2DM is an independent risk factor for osteoporosis (6), can increase the risk of fracture (7). Recently, however, some scholars have suggested that osteoporosis may also be a kind of diabetic microvascular complication (7). Sarcopenia is also an age-related disease (8). It is similar to the mechanism of diabetic microvascular complications, which is mainly related to the expression of oxidative stress pathway (9–13). Musculoskeletal damage includes osteoporosis and sarcopenia, which have similar pathophysiological mechanisms and can worsen each other (14).

In clinical practice, we have observed that patients with T2DM are often accompanied by changes in body weight during the treatment. Other studies have shown that people with early-onset type 2 diabetes (EOT2D) are more obese (15). Combined with the diabetes, this involves a concept called “Metabolic Syndrome”, which includes hyperlipidemia, hyperglycemia, hypertension and abdominal obesity (16). Metabolic syndrome is a common risk factor for osteoporosis, which increases the risk of osteoporosis and fracture in patients with diabetes (16).

Weight gain and obesity have always been considered to be risk factors for diabetes (17). The development of musculoskeletal damage can be regard as a change in body composition. During the treatment of diabetes, we observed changes in body composition in many patients. The relationship between diabetes and musculoskeletal damage has been paid more and more attention by many scholars, and have also been fully studied. But there is no clear point of view on whether there are differences in the changes of musculoskeletal damage and body composition among diabetic people with different age of onset. Therefore, the purpose of this study is to expound the difference of musculoskeletal damage between EOT2D and non-early-onset type 2 diabetes (NOT2D).

## 2 Materials and methods

### 2.1 Research object

This study selected patients who were hospitalized in the Department of Endocrinology, the first affiliated Hospital of Fujian Medical University from April 2008 to November 2020, which is a cross-sectional study, and set the inclusion criteria as follows: (1) patients meeting the diagnostic criteria of T2DM (1); .(2) patients have complete data on body composition and bone mineral density (BMD), and improve the relevant biochemical tests. The exclusion criteria were as follows: (1) patients with type 1 diabetes, gestational diabetes and other special types of diabetes, type 2 diabetic ketoacidosis, type 2 diabetic hyperglycemia and hyperosmotic syndrome, hypoglycemia and lactic acidosis; (2) patients with acute and chronic infection, hepatic and renal insufficiency, cardiac insufficiency, malnutrition and malignant tumor; (3) patients have used or are using anti-osteoporotic drugs such as zoledronic acid and denosumab; (4) excluding the influence of secondary factors (such as hyperthyroidism, parathyroid dysfunction, chronic nephropathy, etc.), and refer to the guidelines for diagnosis and treatment of Primary Osteoporosis (5); (5) pregnant women or athletes. 5176 patients with type 2 diabetes were selected, and the selected patients were classified according to the age of onset. 1:1PSM was performed according to age and sex. Then the original data of 5176 patients with type 2 diabetes were screened according to whether they had two or more hospitalization experiences. Finally, 414 re-admitted patients were obtained, and a cohort study was conducted.

### 2.2 Research methods

#### 2.2.1 Medical history collection and physical examination

By using the patient’s hospitalization number and ID card number for case retrieval, we record the patient’s age, sex, current history, course of type 2 diabetes, age of onset, medication history, past history, personal history and other basic data. and manually proofread it again and record them in the cross-sectional database. For the patients who have been hospitalized at least twice, we judge the order according to the hospitalization time, record the first and last hospitalization records, and check them manually and record them in the cohort database.

All patients underwent a comprehensive physical examination, including but not limited to height, weight, waistline, hip circumference, etc. Measurement of height and weight: getting up in the morning on an empty stomach, after emptying urine, the patient wore light clothes and stood barefoot on the measuring instrument (model: RGZ-120-RT), with both heels together, heel, sacrum and shoulders close to the post of the altimeter. Waist circumference, hip circumference measurement: the patient wears light clothing and the upper limbs are naturally abduction at an angle of about 45 degrees. Take 2 cm above the navel plane and use a soft ruler to surround the skin as closely as possible without pulling the soft ruler; then ask the patient to stand upright with legs together, the upper limbs droop naturally, take the most prominent part of the buttocks, circle around the horizontal plane through the pubic symphysis. Systolic blood pressure (SBP) and diastolic blood pressure (DBP) were measured after resting for at least 15 minutes. BMI equals weight (kg) divided by height squared (m^2^). Waist-to-hip ratio equals waist circumference (cm) divided by hip circumference (cm).

#### 2.2.2 BMD and body composition examination

Dual energy X-ray absorptiometry (GE LUNAR company, prodigy) was used to detect the BMD. The nosocomial standard BMD model is used to correct the BMD of 1.000g/cm2 in the imaging examination room every year. The patients were tested by instrument, and the information of appendicular skeletal muscle mass (ASM), trunk fat mass (TFM), lumbar bone mineral density (L1-4BMD), left femoral neck bone mineral density (FNBMD), left hip joint bone mineral density (Hip-BMD) and android/gynoid ratio (A/G) were measured and recorded. Appendicular skeletal muscle mass index (ASMI) is equal to ASM divided by height^2^ (m^2^). Trunk fat mass index (TFMI) is equal to TFM divided by height^2^ (m^2^). Limb muscle mass/trunk fat mass (A/T) equals ASMI divided by TFMI.

#### 2.2.3 Clinical biochemical examination

All patients fasting, fasting water, and did not get calories from other channels (such as infusion) for more than 8 hours to collect blood in the early morning. We selected glycosylated hemoglobin (HbA1c) (variant II glycosylated hemoglobin analyzer, Bio-Rad, HPLC), total cholesterol (TC), triglyceride (TG), high density lipoprotein cholesterol (HDL-C), low density lipoprotein cholesterol (LDL), creatinine (Cr), serum calcium concentration (Ca^2+^), Serum inorganic-phosphate concentration (P) level (German Siemens ADVIA2400 automatic biochemical analyzer). The glomerular filtration rate (eGFR) of each patient was calculated by Cr. The selection of the formula was determined by the patient’s serum creatinine concentration and sex, as follow: female patients with Cr ≤ 62mmol/L: eGFR=144 × (Cr/62) ^ (- 0.329) × 0.993 ^ (age); female patients with Cr > 62mmol/L: eGFR=144 × (Cr/62) ^ (- 1.209) × 0.993 ^ (age); male patients with Cr ≤ 80mmol/L: eGFR=141 × (Cr/80) ^ (- 0.411) × 0.993 ^ (age). male patients with Cr > 80mmol/L: eGFR=141 × (Cr/80) ^ (- 1.209) × 0.993 ^ (age) (18). Insulin release test was performed to extract and detect the levels of serum C-peptide and blood glucose at the time of 0min, 30min and 120min. Measurement of urinary Microalbumin/creatinine in morning urine samples.

## 3 Diagnosis and definition

### 3.1 Early-onset type 2 diabetes and non-early-onset type 2 diabetes mellitus

According to the age of onset, patients with type 2 diabetes can be divided into patients with early-onset type 2 diabetes (onset age ≤ 40 years old) and non-early-onset type 2 diabetes patients (onset age > 40 years old) (4).

### 3.2 Chronic complications of diabetes mellitus

#### 3.2.1 Diabetic nephropathy

After excluding nephropathy caused by other non-diabetic factors, the diagnostic criteria of diabetic nephropathy (DN) were eGFR < 60mL/min and/or ACR > 30mg/g, lasting for 3 months (19, 20). Because this study is mainly a cross-sectional study, at least one of the patients whose renal function reaches eGFR < 60mL/min and/or ACR > 30mg/g is considered to have diabetic nephropathy. In order to reduce the error, we confirm whether the previous diagnosis of DN is based on the patient’s case data.

#### 3.2.2 Diabetic retinopathy

Regardless of whether the patient has vision loss or not, we recommend that the patient complete the fundus examination. Fundus examination is performed by professional ophthalmologists after mydriasis. Fundus examination after mydriasis showed typical retinal changes, including microhemangioma, hemorrhage and exudate (21).

#### 3.2.3 Diabetic peripheral neuropathy

Possible symptoms of diabetic peripheral neuropathy (DPN) include loss of sensation and numbness or tingling of the lower extremities, tingling or burning pain; these signs may include a decrease in symmetry of the distal sensation or a significant decrease or loss of ankle reflex (22). We use acupuncture to measure pain, 10g nylon for touch, temperature tester for temperature, standard 128Hz tuning fork for vibration, and tendon hammer for ankle reflex (23). This study referred to the clinical diagnostic criteria recommended by the International Conference on Diabetic Peripheral Neuropathy in Toronto in 2009 (22): (1) definite history of diabetes; (2) peripheral neuropathy during or after diagnosis of diabetes; (3) clinical symptoms and signs consistent with the manifestations of DPN; and (4) any one of the following five examinations (acupuncture pain, tactile pressure, temperature, vibration, ankle reflex) in patients with clinical symptoms (pain, numbness, sensory abnormality, etc.). If there are no clinical symptoms, any 2 of the 5 examinations are abnormal, which can be diagnosed as DPN. And neuropathy caused by other causes was excluded at the same time.

#### 3.2.4 Diabetic foot

According to the guidelines of the National Institute of Health and Nursing Excellence, diabetic foot (DF) is defined as an active diabetic foot problem: ulcers, transmitted infections, severe ischemia, gangrene, suspected acute Charcot’s arthropathy, or unexplained fever, redness, swollen feet, or no pain (24).

### 3.3 Sarcopenia

We use the standards developed by the Asian Working Group on Sarcopenia to diagnose sarcopenia when the Appendicular skeletal muscle mass of the patient is corrected by height^2^ (m^2^), male < 7.0kg/m^2^, female < 5.4kg/m^2^ (8, 25). Have study shown that muscle mass is decreasing at a rate of about 2% a year (26). Therefore, we set the change rate of ASM measured in two hospitalizations to be more than 2%, which can be determined as a change in muscle mass. Therefore, patients can be divided into three groups: ASM loss group (ASM <-2%), ASM slight change group (ASM from-2% to 2%), ASM improvement group (ASM > 2%).

### 3.4 Osteoporosis

According to the standards set by the World Health Organization (WHO), osteoporosis means that the BMD of the lumbar vertebrae, hips, femoral neck and distal radius is lower than or above the average 2.5SD of the same age (T-Score ≤-2.5) (5, 27). Studies have shown that the minimum significant change in BMD is set at 3%, that is, a change in BMD of more than 3% is considered to have changed from the previous (28). Therefore, patients can be divided into three groups: BMD loss group (BMD <-3%), BMD slight change group (BMD from-3% to 3%), BMD improvement group (BMD > 3%).

### 3.5 Musculoskeletal damage

Musculoskeletal disease includes three motor system diseases: sarcopenia, osteoporosis and osteoarthritis (14). However, at present, this study only involves sarcopenia and osteoporosis, in order to avoid conflicts in the description of the disease, we use musculoskeletal damage to refer to the presence of sarcopenia and/or osteoporosis.

### 3.6 Statistical analysis

The research data were statistically analyzed using SPSS Statistics 25.0 software (IBM Corp., NY, USA). The normality test was carried out using the single sample Kolmogorov-Smirnov test. The quantitative data with normal distribution were expressed as “average ± standard deviation”, whereas the “median (quartile range)” was used for data with nonnormal distribution. The differences in demographic characteristics, physical examination, and clinical and metabolic parameters of the patients were compared. One-way analysis of variance was used for continuous variables with normal distribution, whereas the rank-sum test was used for continuous variables with nonnormal distribution. Pearson chi-square test (χ^2^ test) was used for classified variables. After adjusting for the confounding factors of diabetic nephropathy, diabetic retinopathy, diabetic peripheral neuropathy, diabetic foot, history of use insulin, history of use biguanide drugs, history of use thiazolidines drugs, history of use antihypertensive drugs, A/G, ACR, TC, TG, HDL, LDL, eGFR, P, Ca, HBA1C and serum C-peptide, linear regression was used to analyze the relationship between onset age and ASMI, TFMI, A/T, A/G. Binary logistic regression was used to analyze the correlation between early-onset diabetes and sarcopenia, osteoporosis, even musculoskeletal damage. And conduct COX survival analysis for patients with at least two hospitalization history, and analyze the correlation between early-onset diabetes, and sarcopenia, osteoporosis and musculoskeletal damage after correcting the above confounding factors. The outliers were rechecked and corrected; otherwise, they were deleted. R studio and R software (version 4.2.3) were used to further analyze the correlation between early-onset diabetes, non-early-onset diabetes and sarcopenia, osteoporosis, musculoskeletal damage; and draw restricted cube bar graph (RCS). A P value < 0.05 indicated a statistically significant difference.

## 4 Results

### 4.1.Demographic characteristics

As shown in **Table 1**, a total of 964 T2DM patients were included in this study, including 534 males and 430 females. The average age was 52 ± 7 years, and the average course of T2DM was 10 ± 8.5 years. Compared with patients with NOT2D, patients with EOT2D were more likely to have sarcopenia, osteoporosis and even musculoskeletal damage (P < 0.05). There were also more patients with EOT2D with hypertension than those with NOT2D (P < 0.05). There was also an above-mentioned trend in drug use (P < 0.05). Similarly, we also observed that the detection rates of DKD, DR, DPN and DF in patients with EOT2D were higher than those in patients with NOT2D (P < 0.05). In the biochemical indexes, it can be seen that the blood lipid level of patients with EOT2D is not as high as the risk of imbalance in patients with NOT2D (P < 0.05). However, in terms of body composition, patients with EOT2D had lower muscle mass and more centrally distributed fat than those with NOT2D (P < 0.05).

**Table 1:** baseline description of 964 patient data after 1:1 PSM*.

	EOT2D (N=482)	NOT2D (N=482)	Total (N=964)	P
Age [year, P50(P25, P75)]	50 (47, 56)	50 (47, 56)	50 (47, 56)	1.000
Male [N (%)]	267 (55.4)	267 (55.4)	534 (55.4)	1.000
Time of onset (year, x ± s)	37 (34, 39)	45 (43, 50)	40 (37, 45)	<0.001
Course of DM (year, x ± s)	14.5 (10, 20)	3 (1, 7)	9 (3, 15)	<0.001
DN [N (%)]	261 (54.1)	154 (32.0)	415 (43.0)	<0.001
DR [N (%)]	195 (40.5)	81 (16.8)	276 (28.6)	<0.001
DPN [N (%)]	262 (54.4)	180 (37.3)	442 (45.9)	<0.001
DF [N (%)]	18 (3.7)	4 (0.8)	22 (2.3)	0.002
Insulin [N (%)]	348 (72.2)	226 (46.9)	574 (59.5)	<0.001
Biguanides [N (%)]	306 (63.5)	253 (52.5)	559 (58.0)	<0.001
Thiazolidines [N (%)]	59 (12.2)	40 (8.3)	99 (10.3)	0.028
Antihypertensive drugs [N (%)]	155 (32.2)	114 (23.7)	269 (27.9)	0.002
Hypertension [N (%)]	222 (46.1)	182 (37.8)	404 (41.9)	0.005
Smoking [N (%)]	123 (25.5)	122 (25.3)	245 (25.4)	0.500
Drinking [N (%)]	56 (11.6)	51 (10.6)	107 (11.1)	0.341
Sarcopenia [N (%)]	209 (43.4)	66 (13.7)	275 (28.5)	<0.001
Osteoporosis [N (%)]	83 (17.2)	45 (9.3)	128 (13.3)	<0.001
Musculoskeletal damage [N (%)]	226 (46.9)	97 (20.1)	323 (33.5)	<0.001
TC [mmol/L, P50(P25, P75)]	4.71 (3.97, 5.60)	5.70 (4.87, 6.68)	5.21 (4.35, 6.16)	<0.001
TG [mmol/L, P50(P25, P75)]	1.44 (0.97, 2.29)	1.73 (1.19, 2.35)	1.59 (1.07, 2.33)	<0.001
HDL [mmol/L, P50(P25, P75)]	1.09 (0.94, 1.37)	1.14 (0.96, 1.38)	1.13 (0.94, 1.37)	0.148
LDL [mmol/L, P50(P25, P75)]	2.94 (2.22, 3.60)	3.87 (3.18, 4.70)	3.38 (2.61, 4.29)	<0.001
eGFR [ml/min, P50(P25, P75)]	106.45 (93.47, 114.13)	108.75 (99.99, 115.95)	107.62 (97.34, 115.11)	0.007
P [mmol/L, P50(P25, P75)]	1.20 (1.10, 1.34)	1.20 (1.09, 1.33)	1.20 (1.09, 1.34)	0.695
Ca^2+^ [mmol/L, P50(P25, P75)]	2.23 (2.14, 2.31)	2.26 (2.18, 2.36)	2.25 (2.16, 2.34)	<0.001
HBA1c [%, P50(P25, P75)]	9.10 (7.60, 10.73)	9.70 (7.60, 11.60)	9.40 (7.60, 11.20)	0.002
0min C-peptide [nmol/L, P50(P25, P75)]	0.51 (0.31, 0.62)	0.56 (0.42, 0.72)	0.54 (0.37, 0.65)	<0.001
30min C-peptide [nmol/L, P50(P25, P75)]	0.76 (0.45, 0.81)	0.78 (0.66, 0.94)	0.77 (0.54, 0.84)	<0.001
120min C-peptide [nmol/L, P50(P25, P75)]	1.49 (0.81, 1.55)	1.52 (1.10, 1.92)	1.51 (0.94, 1.67)	<0.001
ACR [mg/g, P50(P25, P75)]	28.25 (9.25, 164.32)	13.18 (6.87, 33.37)	17.93 (7.80, 73.36)	<0.001
BMI [kg/m^2^, P50(P25, P75)]	24.33 (21.88, 26.68)	24.16 (22.13, 26.57)	24.22 (22.03, 26.57)	0.897
ASMI [kg/m^2^, P50(P25, P75)]	6.63 (5.83, 7.54)	7.03 (6.16, 7.84)	6.81 (6.05, 7.67)	<0.001
TFMI [kg/m^2^, P50(P25, P75)]	3.89 (2.79, 4.86)	3.94 (3.08, 4.91)	3.91 (3.00, 4.89)	0.270
A/T [P50(P25, P75)]	1.77 (1.33, 2.44)	1.76 (1.39, 2.39)	1.76 (1.35, 2.41)	0.683
A/G [P50(P25, P75)]	1.26 (1.11, 1.43)	1.21 (1.03, 1.39)	1.23 (1.07, 1.41)	0.001

*EOT2D, early-onset type 2 diabetes; NOT2D, non-early-onset type 2 diabetes; DM, Diabetes mellitus; DN, Diabetic nephropathy; DR, Diabetic retinopathy; DPN, Diabetic peripheral neuropathy; DF, Diabetic foot; TC, Total cholesterol; TG, Triglyceride; HDL, High density lipoprotein cholesterol; LDL, Low density lipoprotein cholesterol; eGFR, Glomerular filtration rate; P, Inorganic phosphate; Ca^2+^, Calcium; HbA1c, Glycosylated hemoglobin A1c; ACR, Ratio of urinary microalbumin to creatinine; BMI, Body mass index; ASMI, Appendicular skeletal muscle mass index; TFMI, Trunk fat mass index; A/T, Ratio of appendicular skeletal muscle mass to trunk fat mass; A/G, Android gynoid ratio. P < 0.05 indicates that it is statistically significant.

### 4.2 Relationship between age of onset and body composition and BMD

**Figure 1:**
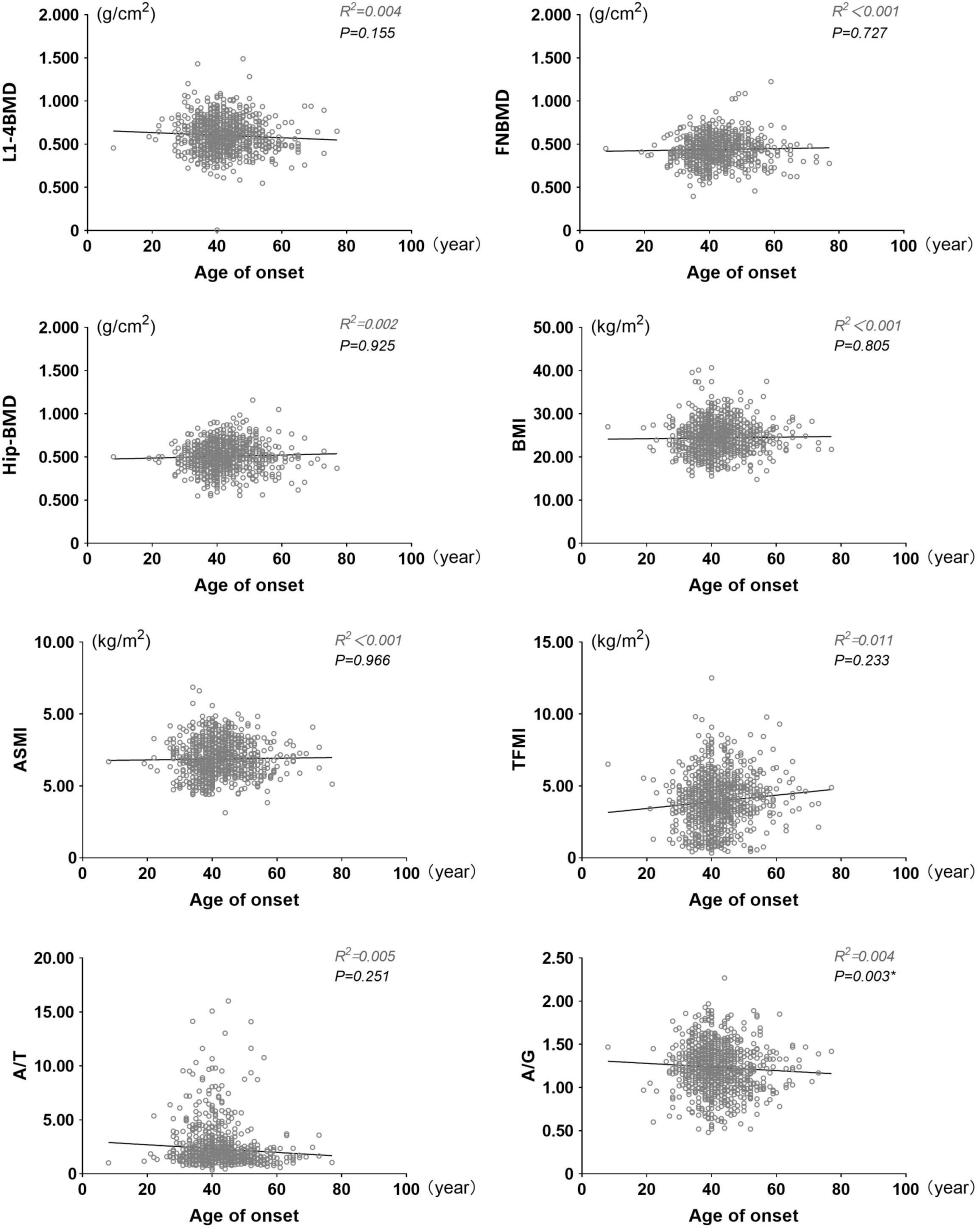
Linear scatter plot between onset age and BMI, ASMI, TFMI, A/T, A/G, L1-4BMD, FNBMD, Hip-BMD. BMI, Body mass index; ASMI, Appendicular skeletal muscle mass index; TFMI, Trunk fat mass index; A/T, Ratio of appendicular skeletal muscle mass to trunk fat mass; A/G, Android gynoid ratio. Adjusted confounding factors: DN, DR, DPN, DF, history of insulin use, history of biguanides use, history of thiazolidines use, history of antihypertensive drugs use, history of hypertension use, ACR, TC, TG, HDL, LDL, eGFR, P, Ca, HbA1c, serum C peptide. P < 0.05 indicates that it is statistically significant.

As shown in **Figure 1**, the age at which diabetes was diagnosed in each patient was recorded and linear regression analysis was performed with BMI, ASMI, TFMI, A/T, A/G, L1-4BMD, FNBMD, Hip-BMD. We corrected some confounding factors (DN, DR, DPN, DF, insulin use history, biguanide use history, thiazolidines use history, antihypertensive drug use history, hypertension history, A/G, ACR, TC, TG, HDL, LDL, eGFR, P, Ca, HbA1c, serum C-peptide). After analysis, it was found that there was a negative correlation between the age of onset and A/G, and there was statistical significance (P=0.003, B=-0.004, 95%CI: -0.006~-0.001). However, there was no significant correlation between the age of onset and BMI, ASMI and TFMI (P > 0.05), but the trend of positive correlation can still be seen from the figure. At the same time, the age of onset was positively correlated with FNBMD, Hip-BMD, but the results were not statistically significant (P > 0.05). FNBMD, It can also be seen in the figure that A/T has a negative correlation with the age of onset, and there is no significant statistical significance (P > 0.05), which may be due to the different correlation between the age of onset and ASMI and TFMI. Similarly, the trend of correlation between L1-4BMD and age of onset is different from that of other sites. This may be caused by the interference of visceral fat, body weight and other factors in the detection of L1-4BMD (**Supplementary Table 1**).

### 4.3 Relationship between EOT2D, NOT2D and musculoskeletal damage

**Figure 2:**
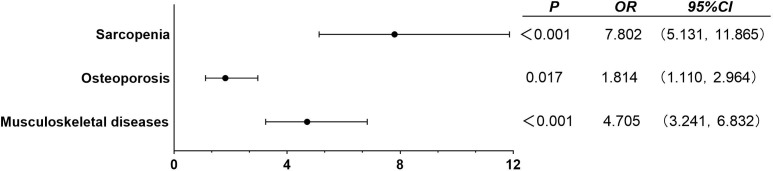
Binary logistic regression analysis of EOT2D, NOT2D and musculoskeletal damage. Adjusted confounding factors: DN, DR, DPN, DF, history of insulin use, history of biguanides use, history of thiazolidines use, history of antihypertensive drugs use, history of hypertension use, ACR, TC, TG, HDL, LDL, eGFR, P, Ca, HbA1c, serum C peptide. P < 0.05 indicates that it is statistically significant.

**Figure 3:**
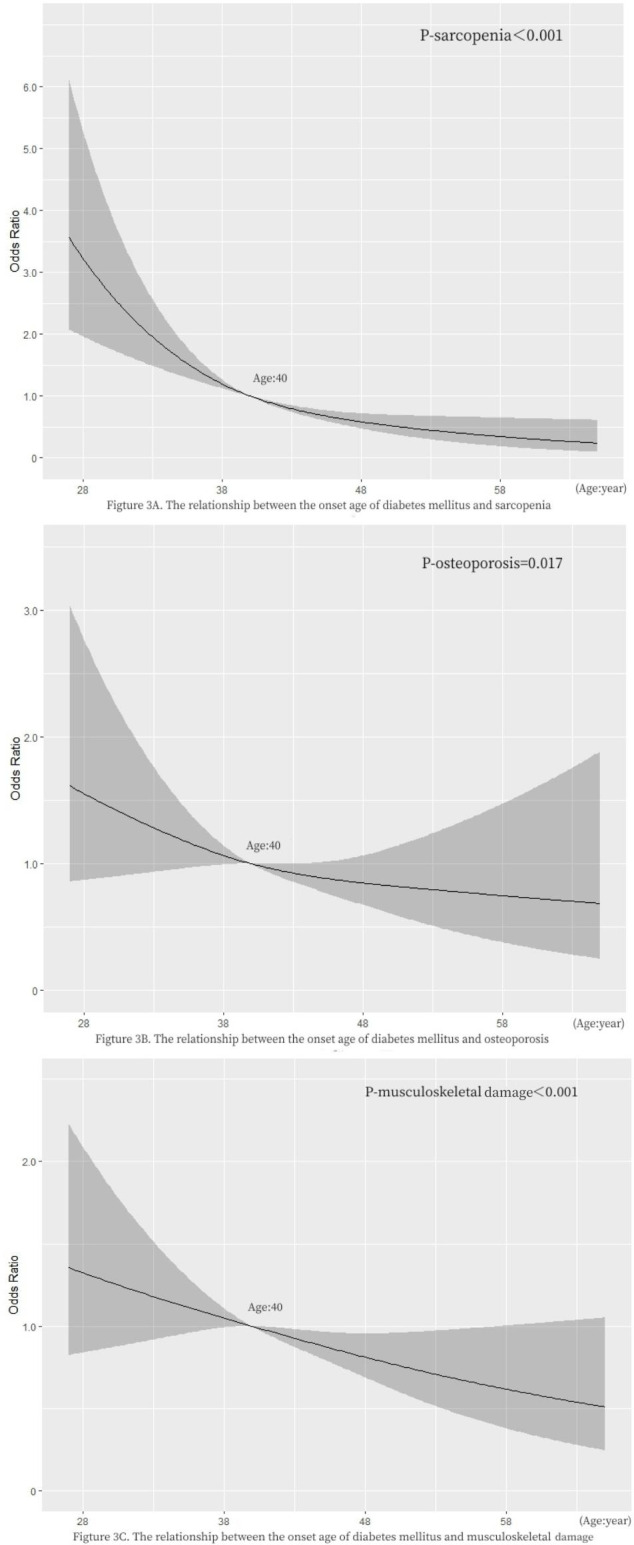
Restricted cubic spline plots of EOT2D, NOT2D and musculoskeletal damage. P < 0.05 indicates that it is statistically significant. The grey part represents the 95% confidence interval of Odds Ratio.

As shown in **Figure 2**, according to the age of onset, diabetic patients are divided into EOT2D and NOT2D (4), then calculate the ASMI and T value according to the patient’s bone mineral density test report to judge whether the patient can be diagnosed as sarcopenia, osteoporosis, or even musculoskeletal damage. After adjusting confounding factors (DN, DR, DPN, DF, history of insulin use, history of biguanides use, history of thiazolidines use, history of antihypertensive drugs use, history of hypertension use, ACR, TC, TG, HDL, LDL, eGFR, P, Ca, HbA1c, serum C peptide), we found: compared with NOT2D, people with EOT2D are at greater risk of developing sarcopenia, osteoporosis and even musculoskeletal damage (sarcopenia: P<0.001, OR= 7.802, 95%CI: 5.131~11.865; osteoporosis: P= 0.017, OR= 1.814, 95%CI: 1.110~2.964; musculoskeletal damage: P<0.001, OR= 4.705, 95%CI: 3.241~6.832). (**Supplementary Table 2**) In addition, we import this data into R software to draw restricted cube splines (**Figure 3**). It can be directly observed from the **Figure 3** that the onset age of diabetes is different, and the risk of sarcopenia, osteoporosis and even musculoskeletal damage is different, but they all show a younger age of onset and a higher risk of sarcopenia, osteoporosis and even musculoskeletal damage (all P < 0.05).

### 4.4 Changes of ASM and BMD in EOT2D and NOT2D

**Figure 4:**
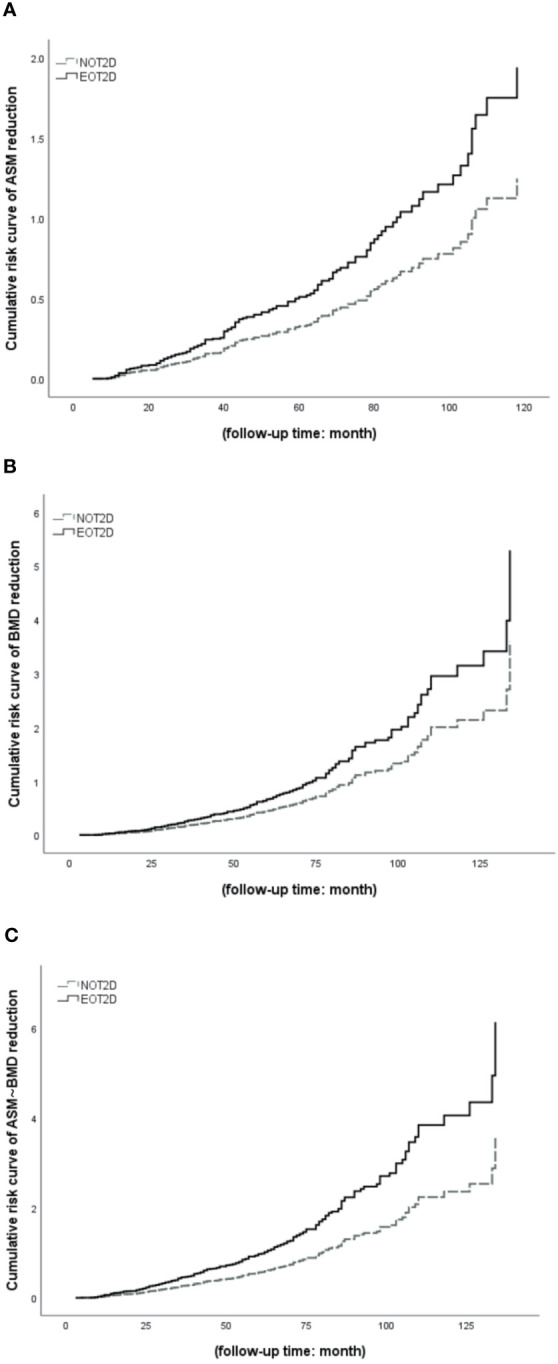
COX survival analysis of EOT2D and NOT2D with the changes of ASM and/or BMD. ASM, appendicular skeletal muscle mass; BMD, bone mineral density; EOT2D, early-onset type 2 diabetes; NOT2D, non-early-onset type 2 diabetes. **(A)** Cumulative risk curve of EOT2D and NOT2D with the changes of ASM. **(B)** Cumulative risk curve of EOT2D and NOT2D with the changes of BMD. **(C)** Cumulative risk curve of EOT2D and NOT2D with the changes of ASM and BMD.

As shown in **Figure 4A–C**, we selected 414 T2DM patients according to whether the patient was hospitalized at least twice. The median follow-up period was 44 months. COX survival analysis was performed on this part of the case data, setting the occurrence of ASM reduction and/or BMD reduction is the final event, which threshold values are respectively 2% and 3%, to further clarify the changes of ASM and/or BMD of EOT2D and NOT2D with the increase of disease course. The results showed that after adjusting for confounding factors (DN, DR, DPN, DF, history of insulin use, history of biguanides use, history of thiazolidines use, history of antihypertensive drugs use, history of hypertension use, ACR, TC, TG, HDL, LDL, eGFR, P, Ca, HbA1c, serum C peptide). With the increase of follow-up time, the cumulative risk of decreased ASM and/or BMD increased in patients with EOT2D and NOT2D, but the risk was higher in patients with EOT2D. and there was statistical significance in the group of ASM change and even ASM-BMD change (ASM decrease: P= 0.032, OR= 1.555, 95%CI: 1.039~2.327; BMD decrease: P= 0.029, OR= 1.473, 95%CI: 1.041~2.085; ASM&BMD decrease: P<0.001, OR= 1.715, 95%CI: 1.271~2.314) (**Supplementary Table 3**).

## 5 Discussion

To the best of our knowledge, this is a rare study that directly discusses the age of onset of diabetes and muscle and bone. In terms of body composition, patients with EOT2D had lower ASM and more centrally distributed fat than those without NOT2D. In terms of diseases, patients with EOT2D were more likely to have sarcopenia, osteoporosis and even musculoskeletal damage. With the increase of follow-up time, the cumulative risk of decreased ASM and/or BMD increased in patients with EOT2D and NOT2D, but the risk was higher in patients with EOT2D.

Diabetes is a disease characterized by elevated blood sugar and affected by various genetic and environmental factors, resulting in progressive loss of islet β-cell mass and/or function (17). High levels of blood glucose promote mitochondrial and cytoplasmic oxidases in vascular endothelial cells, such as NADPH oxidase, which produce excessive reactive oxygen species (ROS) and cause microvascular damage (29). Studies have shown that muscle adipotoxicity can lead to insulin resistance in skeletal muscle cells (12), and oxidative stress is an important pathway leading to sarcopenia (13). These two diseases have similar biochemical mechanisms and are widely distributed in the elderly.

Osteoporosis as an age-related disease (5), which is characterized by low bone mass and degeneration of bone tissue microstructure, resulting in increased bone brittleness, thus increasing the risk of fracture (30). Previous studies have suggested that the increase of BMI is a protective factor of bone mineral density (31), but overweight and even obesity are also important risk factors for diabetes and cardiovascular disease (2, 32).

Sarcopenia is also defined as a disease associated with age-related loss of muscle mass, muscle strength, and muscle function (8). Sarcopenia and T2DM are cause and affect each other. The study of Kalyani et al. (33) shows that the increase of HbA1c is related to the decrease of muscle strength and aging. The study also emphasized that diabetic patients with cognitive impairment had a higher incidence of muscular dystrophy and that blood sugar fluctuations were associated with muscle function. This suggests that blood glucose fluctuations and HbA1c levels are related to muscle mass and function, and continuous monitoring of blood glucose is needed to determine the relationship between blood glucose fluctuations and sarcopenia. Optimal blood glucose control can reduce the prevalence of muscular dystrophy in T2DM patients (34). The treatment of muscular dystrophy includes removing the inducement and improving the etiology. Our guidelines recommend improving bad lifestyle, quitting smoking and drinking, strengthening nutritional support, maintaining good health, exercise, drug intervention and so on (25).

Changes in body composition, or the relationship between body composition and diabetes, osteoporosis and sarcopenia, have attracted more and more attention of scholars. Our previous studies have suggested that the relationship between different BMI and diabetic microvascular complications is different (35). This study showed that with the increase of BMI, the detection rate of DPN and peripheral artery disease (PAD) decreased at first and then increased, while the detection rate of DN and carotid atherosclerotic plaque (CAP) showed an upward trend; however, the change of DR is irregular (35). In addition, other studies evaluated the relationship between BMI, abdominal obesity and FNBMD. In the uncorrected BMI model, waist circumference was positively correlated with FNBMD, while when adjusted for BMI, waist circumference was negatively correlated with FNBMD (36). Although such studies do not directly explore the relationship between specific body composition and osteoporosis and/or sarcopenia, but the weight itself includes muscle, bone, fat and so on, and abdominal obesity refers to the result of fat accumulation in the abdomen. The above studies have demonstrated to varying degrees that the increase of total fat is a risk factor for diabetes and musculoskeletal damage.

The relationship between diabetes, osteoporosis, sarcopenia and body composition changes has been studied by many scholars. However, there are few studies on the relationship between EOT2D and body composition changes or musculoskeletal damage.

Our cross-sectional and follow-up cohort studies have shown that patients with EOT2D may have a higher risk of developing musculoskeletal damage than patients with NOT2D. Cheng Qingfeng et al. (24) systematically studied the relationship between sarcopenia and DF. In their study, patients with sarcopenia had foot ulcers, a higher Wagner grade and a higher amputation rate than patients without sarcopenia, and concluded that sarcopenia was associated with DF. Patients with DF complicating sarcopenia have a poorer prognosis. Compared with DPN and DR, sarcopenia can cause an increase in proteinuria, which has been confirmed by many scholars (37–40). Similarly, other scholars have also discussed the relationship between diabetes and osteoporosis. Chen Hui, Li Xiaoxu and other studies have shown that there is a significant disorder of bone mineral metabolism in patients with T2DM, especially in patients with DN (41). Some studies have shown that there is a negative correlation between microalbuminuria and FNBMD (42). Poor peripheral nerve function may also be directly related to the decrease of BMD (43). The incidence of DR and the detection rate of osteoporosis also increased (44).

In addition, our study also discussed the body composition. Our research shows that the earlier the onset of diabetes, the lower the muscle mass and bone mineral density, and the more fat distribution may tend to be central obesity. This trend is reflected in the A/G ratio, but in this study, there is no significant statistical difference between the onset-age of diabetes and other body composition changes even bone mineral density, but the trend is relatively obvious. Studies have shown that diabetics under the age of 45 are more likely to be obese (15). These studies illustrate the opinion that the younger the onset of diabetes, the greater the proportion of fat in body composition. Our study discussed this more directly and analyzed the fat distribution.

The advantage of this study is that, first of all, we introduce the age of onset and divide type 2 diabetes into EOT2D and NOT2D, instead of just focusing on the occurrence of diabetes, or the relationship between T1DM, T2DM, or other specific types of diabetes and musculoskeletal damage. Secondly, the sample size of our study is large and the time span is long enough, after the PSM, there are still 964 patients. And 414 patients with follow-up data. This makes our data closer to the occurrence and distribution of diseases in the natural population. Third, not only from the diagnosis of osteoporosis and/or sarcopenia to study the relationship between diabetes and musculoskeletal damage, but also from the perspective of body composition, onset age, follow-up time and other angles to understand the relationship between them, and from the cross-section and cohort two different studies and discuss the risk of the disease.

It should be noted that the baseline did not exclude patients with sarcopenia and/or osteoporosis when processing follow-up data in this study, because binary logistic regression analysis showed that patients with EOT2D had a higher risk of developing musculoskeletal damage and that musculoskeletal damage itself, as an age-related disease, increased risk over time. Therefore, simply screening people with related diseases among patients at the baseline level may cause huge errors, which will be difficult to correct and affect the authenticity of the final results. For this reason, we set the changes of ASM and BMD to help determine the effect of the early or late age of onset on muscle and bone. In the linear regression analysis, we can see that the trend of L1-4BMD is different from that of other parts, which may be caused by the dense bone microstructure of lumbar vertebrae caused by the stress of their own body weight for a long time, or by the thickening of abdominal organs and/or fat, which is negatively consistent with the age trend of onset.

Of course, this study also has the following shortcomings. First, all the patients are from inpatients, which will cause group bias, and there may be laboratory errors in different states of the perfect examination. Second, there are still many confounding factors that have not been corrected, such as the daily habits of patients, the history of the use of other drugs, the age of menopause and so on. Third, the division of EOT2D and NOT2D is relatively simple, dichotomy may cause some characteristics to be covered up. Fourth, the course of disease of patients is generally long, and patients may have memory bias in describing their own condition. Therefore, a larger sample size and closer to the real-world study is needed to further explore the relationship between EOT2D and muscle, bone and body composition.

Our study makes a preliminary study on the relationship between EOT2D and musculoskeletal damage and body composition, which is a preliminary description of the relationship between EOT2D and musculoskeletal damage, age of onset and bone density body composition. This may inspire subsequent more complete sample size studies, and provide some ideas and key points for clinical diagnosis and treatment.

In conclusion, EOT2D brings a greater risk of sarcopenia and/or osteoporosis, as well as a higher risk of reduced ASM and BMD. In addition, fat distribution may be more central.

## Data availability statement

The original contributions presented in the study are included in the article/**Supplementary Material**, further inquiries can be directed to the corresponding author/s.

## Ethics statement

The studies involving humans were approved by the ethics committee of the First Affiliated Hospital of Fujian Medical University [MRCTA, ECFAH of FMU (2017)131]. The studies were conducted in accordance with the local legislation and institutional requirements. Written informed consent for participation was not required from the participants or the participants’ legal guardians/next of kin in accordance with the national legislation and institutional requirements.

## Author contributions

BZ: Formal analysis, Writing – review & editing. YZ: Formal analysis, Methodology, Writing – review & editing. LH: Project administration, Writing – original draft. XS: Project administration, Writing – original draft. FZ: Data curation, Writing – review & editing. SY: Writing – original draft, Writing – review & editing.
