# Supplementary material for: Early onset age increases the risk of musculoskeletal damage in patients with type 2 diabetes
Source: Front Endocrinol (Lausanne). 2023 Dec 8;14:1270674. doi: 10.3389/fendo.2023.1270674 (PMC10739489; doi:10.3389/fendo.2023.1270674)
Supplement: Supplementary file 1 [file Table_1.docx]

Supplementary Table 1: Linear scatter plot between onset age and BMI, ASMI, TFMI, A/T, A/G, L1-4BMD, FNBMD, Hip-BMD.

|  | P | R^2^ | B | 95%CI |
| --- | --- | --- | --- | --- |
| Onset age of T2DM | / | / | / | / |
| -L1-4BMD | 0.155 | 0.004 | -0.001 | -0.003~0.001 |
| -FNBMD | 0.727 | ＜0.001 | 0.000 | -0.002~0.001 |
| -Hip-BMD | 0925 | 0.002 | 0.000 | -0.002~0.001 |
| -BMI | 0.805 | ＜0.001 | -0.004 | -0.038~0.030 |
| -ASMI | 0.966 | ＜0.001 | 0.000 | -0.012~0.011 |
| -TFMI | 0.233 | 0.011 | 0.010 | -0.006~0.026 |
| -A/T | 0.251 | 0.005 | -0.010 | -0.028~0.007 |
| -A/G | 0.003 | 0.004 | -0.004 | -0.006~-0.001 |

Note: BMI= Body mass index; ASMI= Appendicular skeletal muscle mass index; TFMI= Trunk fat mass index; A/T = Ratio of appendicular skeletal muscle mass to trunk fat mass; A/G = Android gynoid ratio. Adjusted confounding factors: DN, DR, DPN, DF, history of insulin use, history of biguanides use, history of thiazolidines use, history of antihypertensive drugs use, history of hypertension use, ACR, TC, TG, HDL, LDL, eGFR, P, Ca, HbA1c, serum C peptide. P < 0.05 indicates that it is statistically significant.
