# Supplementary material for: Early onset age increases the risk of musculoskeletal damage in patients with type 2 diabetes
Source: Front Endocrinol (Lausanne). 2023 Dec 8;14:1270674. doi: 10.3389/fendo.2023.1270674 (PMC10739489; doi:10.3389/fendo.2023.1270674)
Supplement: Supplementary file 2 [file Table_2.docx]

Supplementary Table 2: binary logistic regression analysis of EOT2D, NOT2D and musculoskeletal damage.

| Cat. | OR | 95%CI | P |
| --- | --- | --- | --- |
| Sarcopenia |  |  |  |
| - EOT2D | 7.802 | 5.131~11.865 | ＜0.001 |
| - NOT2D | 1 | / | / |
| Osteoporosis |  |  |  |
| - EOT2D | 1.814 | 1.110~2.964 | 0.017 |
| - NOT2D | 1 | / | / |
| Musculoskeletal damages |  |  |  |
| - EOT2D | 4.705 | 3.241~6.832 | ＜0.001 |
| - NOT2D | 1 | / | / |

Note: EOT2D= early-onset type 2 diabetes; NOT2D= non-early-onset type 2 diabetes. Adjusted confounding factors: DN, DR, DPN, DF, history of insulin use, history of biguanides use, history of thiazolidines use, history of antihypertensive drugs use, history of hypertension use, ACR, TC, TG, HDL, LDL, eGFR, P, Ca, HbA1c, serum C peptide. P < 0.05 indicates that it is statistically significant.
