# Supplementary material for: Early onset age increases the risk of musculoskeletal damage in patients with type 2 diabetes
Source: Front Endocrinol (Lausanne). 2023 Dec 8;14:1270674. doi: 10.3389/fendo.2023.1270674 (PMC10739489; doi:10.3389/fendo.2023.1270674)
Supplement: Supplementary file 3 [file Table_3.docx]

Supplementary Table 3: COX survival analysis of EOT2D and NOT2D with the changes of ASM and/or BMD.

|  | EOT2D | | NOT2D | | Total | | P | OR | 95%CI |
| --- | --- | --- | --- | --- | --- | --- | --- | --- | --- |
|  | baseline | Revisit | baseline | Revisit | baseline | Revisit |  |  |  |
| Age (year） | 47±8 | 50(45, 55) | 63(56, 69) | 66.72±9.31 | 60(51, 68) | 64(55, 71) |  |  |  |
| Male | 51(62.2) | 51(62.2) | 183(55.1) | 183(55.1) | 234(56.5) | 234(56.5) |  |  |  |
| Time of onset (year） | 37(33, 39) | 37(33, 39) | 54(47, 60) | 53(47, 60) | 51(42,58) | 51(43, 59) |  |  |  |
| Course of DM (year） | 10.0(4.0, 17.3) | 14.0(7.0, 20.0) | 8.0(3.0, 11.0) | 10.0(8.0, 16.0) | 8.0(3.0,12.0) | 11.0(8.0, 18.0) |  |  |  |
| DN | 27(32.9) | 33(40.2) | 56(16.9) | 110(33.1) | 83(20.0) | 143(34.5) |  |  |  |
| DR | 20(24.4) | 28(34.1) | 51(15.4) | 87(26.2) | 71(17.1) | 115(27.8) |  |  |  |
| DPN | 35(42.7) | 45(54.9) | 159(47.9) | 212(63.9) | 194(46.9) | 257(62.1) |  |  |  |
| DF | 4(4.9) | 2(2.4) | 7(2.1) | 7(2.1) | 11(2.7) | 9(2.2) |  |  |  |
| Insulin | 49(59.8) | 58(70.7) | 163(49.1) | 216(65.1) | 212(51.2) | 274(66.2) |  |  |  |
| Biguanides | 52(63.4) | 60(73.2) | 214(64.5) | 244(73.5) | 266(64.3) | 304(73.4) |  |  |  |
| Thiazolidines | 14(17.1) | 11(13.4) | 43(13.0) | 33(9.9) | 57(13.8) | 44(10.6) |  |  |  |
| Antihypertensive drugs | 13(15.9) | 35(42.7) | 106(31.9) | 189(56.9) | 119(28.7) | 224(54.1) |  |  |  |
| Hypertension | 41(50.0) | 51(62.2) | 211(63.6) | 249(75.0) | 252(60.9) | 300(72.5) |  |  |  |
| Smoking | 17(20.7) | 21(25.6) | 61(18.4) | 85(25.6) | 78(18.8) | 106(25.6) |  |  |  |
| Drinking | 9(11.0) | 11(13.4) | 18(5.4) | 31(9.3) | 27(6.5) | 42(10.1) |  |  |  |
| Sarcopenia | 26(31.7) | 19(23.2) | 74(22.3) | 88(26.5) | 100(24.2) | 107(25.8) |  |  |  |
| Osteoporosis | 13(15.9) | 5(6.1) | 68(20.5) | 55(16.6) | 81(19.6) | 60(14.5) |  |  |  |
| Musculoskeletal damage | 29(35.4) | 22(26.8) | 122(36.7) | 129(38.9) | 151(36.5) | 151(36.5) |  |  |  |
| TC (mmol/L) | 4.73±1.11 | 4.47(3.73, 5.03) | 4.59(3.85, 5.47) | 4.16(3.48,5.07) | 4.59(3.87, 5.46) | 4.24(3.53, 5.06) |  |  |  |
| TG (mmol/L) | 1.85(1.02, 2.46) | 1.46(1.08, 2.29) | 1.39(0.96, 2.16) | 1.34(0.91,1.96) | 1.47(0.98, 2.25) | 1.37(0.94, 2.00) |  |  |  |
| HDL (mmol/L) | 1.06(0.91, 1.36) | 1.03(0.91, 1.30) | 1.11(0.93, 1.32) | 1.03(0.86, 1.24) | 1.10(0.93, 1.33) | 1.03(0.87, 1.24) |  |  |  |
| LDL (mmol/L) | 2.79±1.00 | 2.76±0.97 | 2.87±0.98 | 2.59(1.92, 3.30) | 2.85±0.99 | 2.63(1.93, 3.30) |  |  |  |
| eGFR (ml/min) | 109.79(102.43, 118.12) | 108.04(100.66, 113.46) | 96.98(86.76, 107.83) | 92.07(77.53, 102.22) | 99.24(88.47, 109.67) | 95.82(79.53, 105.52) |  |  |  |
| P (mmol/L) | 1.21±0.24 | 1.21±0.19 | 1.13(1.01, 1.28) | 1.15±0.21 | 1.14(1.02, 1.29) | 1.15(1.03, 1.28) |  |  |  |
| Ca^2+^ (mmol/L) | 2.24±0.12 | 2.24±0.13 | 2.22(2.14, 2.32) | 2.22(2.14, 2.30) | 2.23(2.15, 2.32) | 2.23(2.14, 2.31) |  |  |  |
| HBA1c (%) | 9.12±2.24 | 8.30(6.70, 10.10) | 8.86(7.30, 11.00) | 8.20(6.90, 9.70) | 8.86(7.39, 10.83) | 8.20(6.80, 9.73) |  |  |  |
| 0min C-peptide (nmol/L) | 0.52(0.35, 0.65) | 0.55(0.39, 0.62) | 0.58(0.38, 0.66) | 0.58(0.47, 0.67) | 0.57(0.38, 0.65) | 0.57(0.45, 0.66) |  |  |  |
| 30min C-peptide (nmol/L) | 0.78(0.59, 0.87) | 0.77(0.55, 0.85) | 0.81(.62, 0.93) | 0.80(0.64, 0.90) | 0.80(0.62, 0.91) | 0.79(0.63, 0.89) |  |  |  |
| 120min C-peptide (nmol/L) | 1.54(1.21, 1.63) | 1.51(0.98, 1.57) | 1.56(1.04, 1.80) | 1.55(1.24, 1.70) | 1.55(1.06, 1.78) | 1.55(1.14, 1.66) |  |  |  |
| ACR (mg/g) | 12.90(5.55, 89.83) | 21.98(6.57, 268.29) | 15.37(6.91, 181.56) | 37.83(8.38, 301.07) | 15.28(6.73, 145.07) | 33.78(7.87, 301.07) |  |  |  |
| BMI (kg/m^2^) | 25.23(23.34, 27.00) | 25.46±3.26 | 24.62±3.13 | 24.71±3.30 | 24.77±3.24 | 24.86±3.30 |  |  |  |
| ASMI (kg/m^2^) | 7.35±1.27 | 7.18±1.26 | 6.81±0.97 | 6.82±0.98 | 6.92±1.06 | 6.89±1.05 |  |  |  |
| TFMI (kg/m^2^) | 4.07±1.57 | 4.40±1.49 | 4.13±1.63 | 4.36±1.74 | 4.12±1.62 | 4.36±1.69 |  |  |  |
| A/T | 1.85(1.42, 2.38) | 1.71(1.30, 2.10) | 1.73(1.25, 2.33) | 1.56(1.20, 2.22) | 1.75(1.29, 2.34) | 1.58(1.21, 2.17) |  |  |  |
| A/G | 1.26(1.18, 1.43) | 1.31±0.23 | 1.25(1.23, 1.28) | 1.24(1.11, 1.34) | 1.25(1.23, 1.28) | 1.25(1.12, 1.38) |  |  |  |
| ASM reduction |  |  |  |  |  |  | 0.032 | 1.555 | 1.039~2.327 |
| ASM increase |  |  |  |  |  |  | / | / | / |
| BMD reduction |  |  |  |  |  |  | 0.029 | 1.473 | 1.041~2.085 |
| BMD increase |  |  |  |  |  |  | / | / | / |
| ASM~BMD reduction |  |  |  |  |  |  | ＜0.001 | 1.715 | 1.271~2.314 |
| ASM~BMD increase |  |  |  |  |  |  | / | / | / |

Note: EOT2D=early-onset type 2 diabetes; NOT2D=non-early-onset type 2 diabetes; TC= Total cholesterol; TG= Triglyceride; HDL= High density lipoprotein cholesterol; LDL= Low density lipoprotein cholesterol; eGFR= Glomerular filtration rate; P = Inorganic phosphate; Ca^2+^ = Calcium; HbA1c= Glycosylated hemoglobin A1c; ACR= Ratio of urinary microalbumin to creatinine; BMI= Body mass index; ASMI= Appendicular skeletal muscle mass index; TFMI= Trunk fat mass index; A/T = Ratio of appendicular skeletal muscle mass to trunk fat mass; A/G = Android gynoid ratio. The normality test was carried out using the single sample Kolmogorov-Smirnov test. The quantitative data with normal distribution were expressed as “average ± standard deviation”, whereas the “median (quartile range)” was used for data with nonnormal distribution. P < 0.05 indicates that it is statistically significant.
